# Comparison of Kato–Katz, PCR and coproantigen for the diagnosis of *Taenia solium* taeniasis

**DOI:** 10.1017/S0031182023000690

**Published:** 2023-09

**Authors:** Marshall W. Lightowlers, Diana Edithe Andria Mananjara, Mihajamanana Rakotoarinoro, Valisoa C. Rakotoarison, Modestine Raliniaina, Harentsoaniaina Rasamoelina-Andriamanivo, Charles G. Gauci, Abdul Jabbar, Kabemba E. Mwape, Meritxell Donadeu, Noromanana Sylvia Ramiandrasoa, Jose Alphonse Nely

**Affiliations:** 1 Department of Biosciences, Melbourne Veterinary School, University of Melbourne, Werribee, Victoria 3030, Australia; 2 National Center for Applied Research on Rural Development (FOFIFA), Antananarivo 101, Madagascar; 3 Indian Ocean Commission/SEGA-One Health Network, Ébène, Mauritius; 4 Department of Clinical Studies, School of Veterinary Medicine, University of Zambia, Lusaka 10101, Zambia; 5 Initiative for Neglected Animal Diseases (INAND), Pretoria, South Africa; 6 Consultant, Antananarivo 102, Madagascar; 7 Ministry of Health of Madagascar, Tananarive 101, Madagascar

**Keywords:** coproantigen, coprology, diagnosis, eggs, Kato–Katz, PCR, *Taenia solium*, taeniasis

## Abstract

Four methods were compared for the diagnosis of human taeniasis caused by *Taenia solium.* Fecal samples from persons living in a *T. solium* endemic region of Madagascar were examined for taeniid eggs by the Kato–Katz method. Subsequently, samples positive (*n* = 16) and negative (*n* = 200) for *T. solium* eggs were examined by (i) amplification of the fragment of small subunit of the mitochondrial ribosomal RNA (*rrn*S) gene using conventional polymerase chain reaction (PCR) and (ii) a nested PCR of a fragment of the *T. solium Tso31* gene. Additionally, 12 egg-positive and all egg-negative samples were tested for coproantigen detection. A further 9 egg-positive fecal samples were examined using both PCRs. Of the 12 egg-positive samples tested by PCRs and coproantigen methods, 9 (75%) were positive by *rrn*S PCR, 3 (25%) using *Tso31*-nested PCR and 9 (75%) by coproantigen testing. None of the 200 egg-negative fecal samples was positive in either *rrn*S or *Tso31*-nested PCR. Twenty of the 25 egg-positive samples (80%) were positive in *rrn*S PCR, and DNA sequencing of PCR amplicons was obtained from 18 samples, all confirmed to be *T. solium*. Twelve of the 25 egg-positive samples (48%) were positive in the *Tso31*-nested PCR, all of which were also positive by *rrn*S PCR. It is suggested that species-specific diagnosis of *T. solium* taeniasis may be achieved by either coprological examination to detect eggs or coproantigen testing, followed by *rrn*S PCR and DNA sequencing to confirm the tapeworm species in egg-positive or coproantigen-positive samples.

## Graphical abstract

**Figure:**
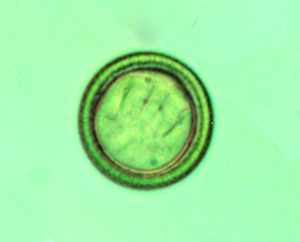

## Introduction

*Taenia solium* is the aetiological agent of neurocysticercosis in humans, one of a number of neglected tropical diseases recognized by the World Health Organization (2015). The parasite is transmitted in a cycle between humans, who harbour the adult tapeworm in the small intestine (taeniasis), and pigs where the larval stage (cysticercus) develops in the muscles and brain after ingesting feces or other items contaminated with *T. solium* eggs. Humans may also develop cysticercosis by ingesting eggs from the feces of a person harbouring the *T. solium* tapeworm. Infection in the brain and other nervous tissue of humans by *T. solium* cysts (neurocysticercosis) is a serious cause of morbidity in areas having poor sanitation and free-roaming pigs (Garcia *et al*., 2020).

Efforts to prevent the transmission of *T. solium* and thereby reduce the incidence of neurocysticercosis rely on the treatment of patients with taeniasis, vaccination and medication of pigs, and improvements in sanitation and pig-rearing practices (Lightowlers, 2013).

Diagnostic tests for taeniasis are undertaken to determine the risk for transmission of cysticercosis in humans, identify endemic areas and to determine the outcomes of control programmes. *Taenia solium* taeniasis can be diagnosed by detection in the feces of eggs, tapeworm segments, parasite antigens or *T. solium* DNA in the feces (Praet *et al*., 2013), or by serology with recombinant antigens (Levine *et al*., 2007).

Human taeniasis is caused by 3 *Taenia* spp., including *T. solium*, *T. saginata* and *T. asiatica*. However, only *T. solium* causes neurocysticercosis and warrants a public health intervention. *Taenia saginata*, in particular, is widely distributed in areas where *T. solium* is prevalent; hence, diagnostic tests for *T. solium* taeniasis must differentiate *T. solium* from infection with other *Taenia* species. Egg morphology does not allow differentiation among *Taenia* species. Similarly, the coproantigen tests that have been described and well validated are unable to differentiate between *Taenia* spp. Recombinant antigens required to undertake species-specific serology (Levine *et al*., 2007) are not readily available. Serological methods are also limited because they are unable to differentiate a current infection from past infection. At least 20 different DNA-based methods have been described for species-specific differentiation of *T. solium*, including at least 15 different polymerase chain reaction (PCR)-based methods; however, few have been validated carefully with parasitologically proven fecal samples from patients with *T. solium* taeniasis (Lightowlers *et al*., 2016).

Here we compare 4 tests for the diagnosis of taeniasis and evaluate coproantigen and 2 PCR-based tests for species-specific diagnosis of *T. solium* taeniasis.

## Methods

### Human fecal samples

Human fecal samples were collected from people in a contiguous area of Betafo and Mandoto provinces of Madagascar as part of baseline evaluations for a *T. solium* control programme. The region was known to be endemic for *T. solium*. Two fecal samplings were undertaken. Initially, 960 samples were collected from randomly selected individuals. Samples were examined freshly for the presence of eggs and aliquots of approximately 2 g were suspended in a 10× volume of 90% ethanol (for DNA analyses) and stored at room temperature. Subsequently, a further 960 fecal samples were selected by purposive sampling, selecting random individuals proportional to the number of pigs present in the area, instead of proportional to the number of people as it was done in the first sampling. These samples were examined and stored in ethanol as above, and in addition, a 2 g sample was placed into a 10× volume of 10% formalin (for coproantigen testing) and stored at room temperature. A further 9 egg-positive fecal samples stored in ethanol were available from a previous study undertaken in Madagascar (Ramiandrasoa *et al*., 2020).

### Egg detection

All fecal samples were examined using the Kato–Katz technique to identify taeniid eggs (Katz *et al*., 1972). Most of the samples were evaluated using 1 slide for the Kato–Katz; however, during the second sampling, 78 samples were evaluated by using 2 slides from the same sample.

### DNA isolation

Fecal DNA was isolated from all egg-positive fecal samples (*n* = 25) as well as from 200 randomly selected fecal samples which were found to be egg-negative. Approximately, 250 mg feces were placed in a 1.5 mL microtube and centrifuged at 1000 ***g*** for 1 min. The supernatant was discarded and the pellet was resuspended in 1 mL of distilled water by vortexing. Following re-centrifugation, the pelleted feces were processed for DNA isolation using the Qiagen QIAamp® PowerFecal® Pro DNA kit and Qiagen TissueLyser II homogenizer, according to the manufacturer's instructions with elution of fecal DNA in a volume of 50 μL. Purified DNA quantities were determined using the NanoDrop One instrument (ThermoFisher Scientific, Wilmington, DE, USA).

### *rrn*S PCR

All egg-positive and 200 egg-negative fecal DNA samples were assessed by *rrn*S PCR. A 267 bp fragment of the small subunit of the mitochondrial ribosomal RNA was amplified, corresponding to positions 12 208–12 475 on the complete mitochondrial genome of *T. solium* (GenBank AB086256.1). The PCR was based on the generic *Taenia* spp. PCR described by Trachsel *et al*. (2007) for the investigation of *Taenia* spp. infecting canines and also used by Ash *et al*. (2017) for human taeniasis. Modified primers (hCest3 5ʹ TGA TTC TTT TTA GGG GAA GGT GTR GTG 3ʹ, hCest5 5ʹ GCG GTG TGT ACA TGA GYT AAA C 3ʹ) were designed more specifically to suit amplification of the sequences from the 3 *Taenia* sp. infecting humans. Magnesium ion concentration and annealing temperature in PCR were optimized using purified *T. solium* genomic DNA (Gauci *et al*., 2019). Fifty microlitre reaction volumes were prepared containing 3 mm MgCl_2_, 50 μm deoxynucleotide triphosphate (dNTP) (Promega), 0.5 μm hCest3 and hCest5 primers, GoTaq Green Reaction Buffer (Promega), 1.25 U GoTaq Flexi DNA polymerase (Promega) and, unless otherwise noted, 2 μL isolated fecal DNA. Controls included 40 pg purified genomic DNA from *T. solium*, *T. saginata* and/or *T. asiatica* (Gauci *et al*., 2019), and fecal DNA isolated from a volunteer known never to have been infected with *Taenia* sp. PCR conditions were 94°C for 5 min followed by 35 cycles of 94°C for 30 s, 58°C for 30 s, 72°C for 30 s, followed by the final extension at 72°C for 5 min.

### Agarose electrophoresis and DNA sequencing

PCR products were electrophoresed in 1.5% agarose, 0.5× Tris-borate-ethylenediaminetetraacetic acid (EDTA) buffer (0.05 m Tris, 0.05 m boric acid, 0.01 m EDTA), 1:10 000 Gel Red (Biotium). PCRs in which a 267 bp *rrn*S product could be detected in agarose electrophoresis were processed for DNA sequencing. Briefly, 10 μL of PCR amplicon were treated with 20 U exonuclease 1 (ThermoFisher) and 2 U shrimp alkaline phosphatase (ThermoFisher) at 37°C for 30 min and 85°C for 15 min. The DNA sequences of PCR amplicons were determined using the hCEST5 primer by Macrogen, North Korea, using Sanger sequencing. NCBI BLAST comparisons of *Taenia* spp. DNA sequences amplified from fecal DNA with the appropriate segment of *T. solium* (GenBank AB086256.1), *T. saginata* (GenBank AY684274.1) or *T. asiatica* (GenBank AP017670.1) mitochondrial DNA sequences readily allowed species differentiation.

### *Tso31*-nested PCR

Egg-positive (*n* = 25) and 200 egg-negative fecal DNA samples were assessed by nested PCR amplifying the single copy of the *T. solium* genomic gene *Tso31* as previously described by Mayta *et al*. (2008). Initially, precisely the same commercial reagent suppliers and conditions described by Mayta *et al*. (2008) were used. The same nested PCR was undertaken using reagent sources as indicated above for the *rrn*S PCR and no improvement was found through the use of the particular reagent sources used by Mayta *et al*. (2008). For that reason, the following reagents and conditions were adopted for use in 50 μL *Tso31*-nested PCR reactions. Outer PCR: 3 mm MgCl_2_, 50 μm dNTP (Promega), 0.5 μm F1 primers (5ʹ ATG ACG GCG GTG CGG AAT TCT G 3ʹ) and R1 primer (5ʹ TCG TGT ATT TGT CGT GCG GGT CTA C 3ʹ) and 4 μL fecal DNA. Incubations were 95°C for 3 min followed by 25 cycles of 95°C for 30 s, 55°C for 30 s and 72°C for 1 min. The inner PCR used similar procedures with 2 μL of the outer PCR reaction, except for an MgCl_2_ concentration of 2.5 mm and 40 cycles, using primers F589 (5ʹ GGT GTC CAA CTC ATT ATA CGC TGT G 3ʹ) and R294 (5ʹ GCA CTA ATG CTA GGC GTC CAG AG 3ʹ). PCR amplicons were analysed on an agarose gel as described above.

### Coproantigen

The stool samples stored in 10% formalin were examined for coproantigens using the polyclonal antibody-based enzyme-linked immunosorbent assay as described by Allan *et al*. (1990) with slight modifications (Mwape *et al*., 2012). To determine the test result, the optical density of each stool sample was compared with the mean of a series of 8 reference *Taenia*-negative stool samples plus 3 standard deviations (cut-off).

## Results

### Egg detection

Four (0.4%) of the 960 fecal samples randomly selected from the study population were found to have *Taenia* eggs present using the Kato–Katz method. A further 12 fecal samples were found to have *Taenia* eggs present among the 960 samples obtained by purposive sampling (1.25%). All the egg-positive samples were identified as positive among the samples tested using a single slide, except for one, which was identified among the 78 samples tested by 2 slides, and was positive in both slides.

### Species-specific diagnosis of *T. solium* taeniasis

A total of 25 fecal samples from different people, which were found to be positive for *Taenia* spp. eggs by Kato–Katz, were examined using *rrn*S and *Tso31*-nested PCRs, as well as 200 randomly selected egg-negative fecal samples. The concentration of DNA obtained from fecal samples using the PowerFecal® Pro DNA kit ranged from 0.1 to 120.1 ng μL^−1^, depending on the organic content of the fecal sample. Twenty of the 25 egg-positive samples (80%) were positive in *rrn*S PCR. Of those 20 samples, the DNA sequence of the PCR product was successfully obtained from 18 samples, all of which were confirmed to be *T. solium* (GenBank accession no. OR098460). Twelve of the 25 egg-positive samples (48%) were positive in the *Tso31*-nested PCR, all of which were also positive by *rrn*S PCR.

### Analytical sensitivity and specificity of the *Tso31* PCR

**Figure 1.**
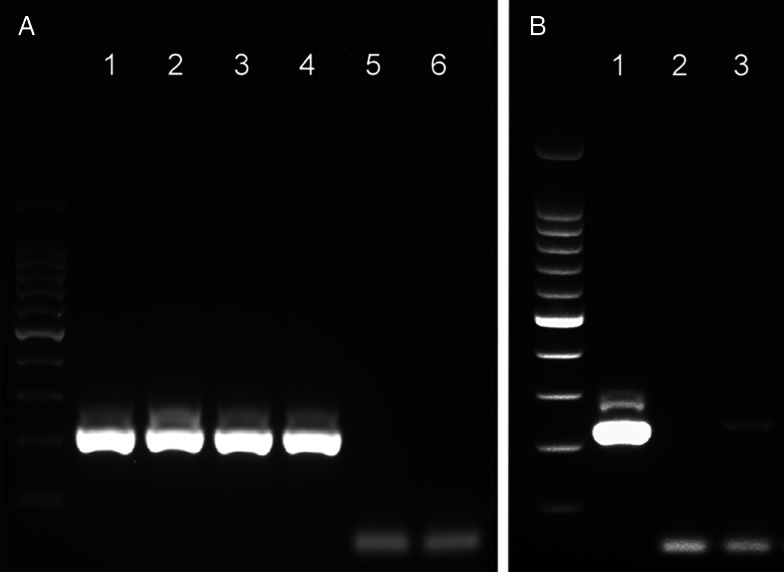
Agarose gel electrophoresis of PCR products showing analytical sensitivity and specificity of the *Tso31*-nested PCR. (A) Titration of *T. solium* DNA in PCR; 100 bp markers, lanes 1–6 PCRs containing 10 pg, 5 pg, 1 pg, 500 fg, 200 fg, 100 fg, DNA respectively. (B) PCR with different *Taenia* sp. DNA; 100 bp markers, lane 1 *T. solium*, lane 2 *T. saginata*, lane 3 *T. asiatica*.

The analytical sensitivity of the *Tso31*-nested PCR was determined using purified *T. solium* genomic DNA (Fig. 1A) for comparison with the data published by Mayta *et al*. (1998). Positive reactions were seen using 500 fg DNA but not at 200 fg. Specificity of the assay is demonstrated in Fig. 1B. No reaction products were found with *T. saginata* genomic DNA; however, a weak band was evident with genomic DNA from *T. asiatica*. This band was sequenced using the F589 primer which revealed a sequence (GenBank OQ476203) having 93% identity with the corresponding segment of the *Tso31*-nested gene.

### Sensitivity comparison of PCRs with fecal DNA

**Figure 2.**
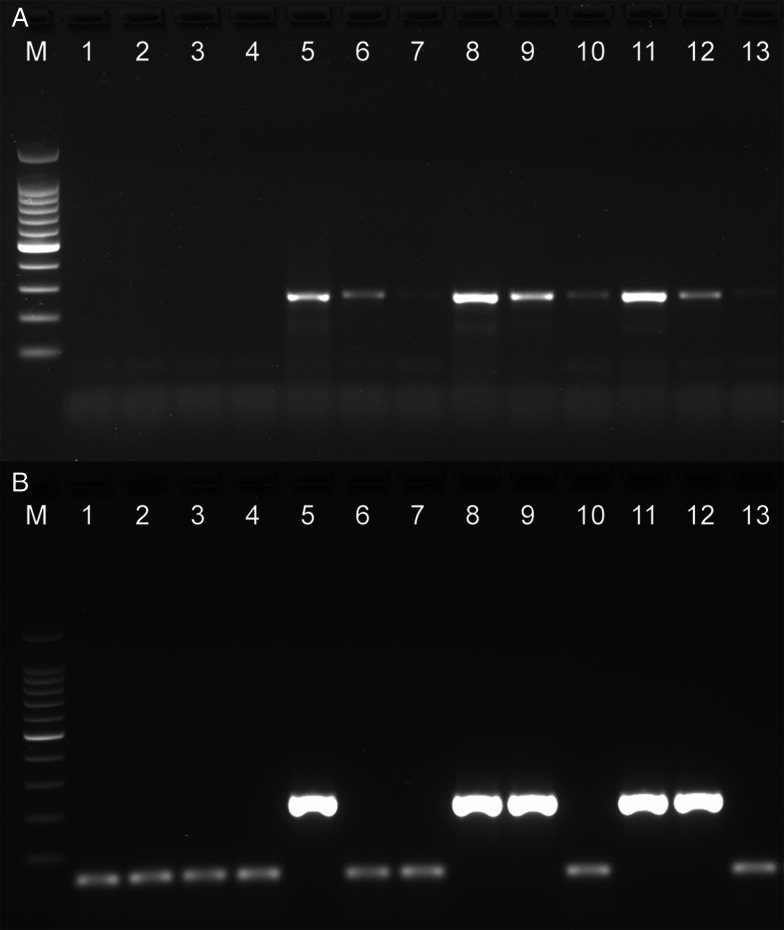
Comparative sensitivity of *rrn*S PCR (A) and *Tso31*-nested PCR (B) using dilutions of DNA isolated from fecal samples. 100 bp markers, lane 1 distilled water, lanes 2–4 fecal DNA from a known taeniasis-negative individual undiluted (2 μL), 1:10 dilution and 1:100 dilution, respectively; lanes 5–7, 8–10, 11–13 dilutions of fecal DNA from 3 fecal samples with proven *T. solium* infection, undiluted (2 μL), 1:10 dilution and 1:100 dilution, respectively.

The *rrn*S and *Tso31*-nested PCRs were compared using dilutions of DNA isolated from the feces of proven *T. solium* tapeworm carriers. Figure 2 shows the results of *rrn*S PCR (top panel) and *Tso31*-nested PCR (bottom panel) using the same DNA samples. DNA from egg-positive fecal samples remained detectably positive in *rrn*S PCR at 1:10 and 1:100 dilutions of the fecal samples; however, there was an absence of detectable product in some of the same dilution samples using the *Tso31*-nested PCR (Fig. 2).

### Diagnostic sensitivity comparison of egg detection, PCR and coproantigen

**Figure 3.**
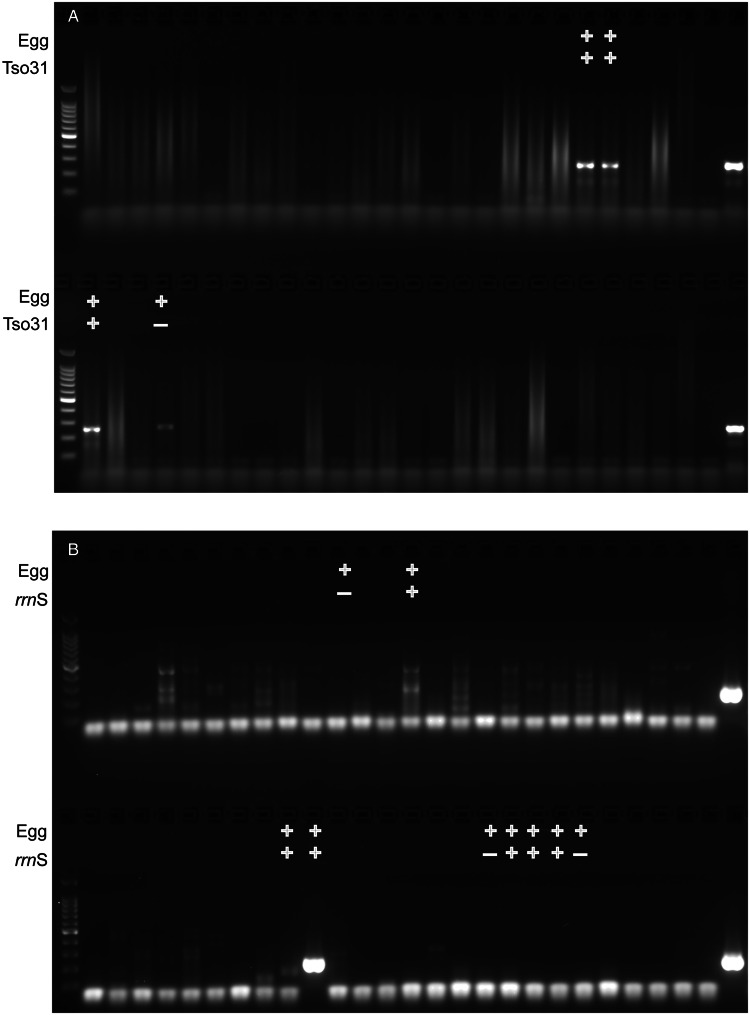
Agarose gel electrophoresis of (A) *rrn*S PCR and (B) *Tso31*-nested PCR products obtained from DNA isolated from a subset of 52 human fecal samples that were tested by each method (different fecal samples shown in A and B). Egg+ indicates the fecal sample was positive for taeniid eggs by Kato–Katz. *Tso31*+ indicates that specific sample tested positive by *Tso31*-nested PCR, *rrn*S+ indicates that specific sample tested positive by *rrn*S PCR. Left hand side lanes, 100 bp markers; right hand side lanes, *T. solium* DNA positive control.

The comparative diagnostic performance of PCR and coproantigen detection for the diagnosis of taeniasis is detailed in Table 1 for those samples for which *rrn*S PCR, *Tso31*-nested PCR and coproantigen testing were performed on each. Of the 12 egg-positive samples, 9 (75%) were positive by *rrn*S PCR, 3 (25%) were positive by *Tso31*-nested PCR and 9 (75%) were positive by coproantigen testing. Example results obtained in *rrn*S PCR and the *Tso31*-nested PCR are shown in Fig. 3. None of the 200 egg-negative fecal samples was positive in either *rrn*S or *Tso31*-nested PCR. One egg-positive sample was negative in all other tests and 2 egg-positive samples (8%) that were PCR-negative were found to be positive by coproantigen testing. Two egg-negative samples were positive by coproantigen testing.

**Table 1.** Comparison of the diagnostic performance of *rrn*S PCR, *Tso31*-nested PCR and coproantigen tests for the diagnosis of taeniasis in 12 fecal samples from different persons which were egg-positive for *Taenia* spp. by the Kato–Katz method. In addition, 200 egg-faecal samples which were negative for *Taenia* spp. eggs by Kato-Katz were also tested.

KK	*rrn*S PCR	*Tso31-*nested PCR	CoproAg	Number	% of KK positives
+	+	+	+	1	8
+	−	+	+	0	
+	+	−	+	6	50
+	+	+	−	2	16
+	+	−	−	0	
+	−	+	−	0	
+	−	−	+	2	16
+	−	−	−	1	8
−	−	−	+	2	
−	−	−	−	198	

KK, Kato–Katz test; CoproAg, coproantigen test; % of KK positives, percentage of all samples positive in Kato–Katz test.

## Discussion

Results of the detection of eggs in the feces by the Kato–Katz method corresponded with those of most egg-positive samples tested by *rrn*S PCR (Table 1). Of 12 fecal samples in which eggs were detected by Kato–Katz, 9 (75%) were positive in *rrn*S PCR. Of 9 fecal samples from proven cases of *T. solium* taeniasis (by egg detection and *rrn*S DNA sequencing), 7 (78%) were positive by coproantigen. Two of the 12 egg-positive samples were positive by coproantigen but not by PCR. Coproantigen testing identified 2 positive samples among the 200 egg-negative feces which were negative by PCR. It is unknown whether these samples were false positives in the coproantigen test or false negatives in Kato–Katz and PCR. Coproantigens can be detected in fecal samples collected prior to worm patency in both human (Tembo and Craig, 2015) and animal (Deplazes *et al*., 1990; Allan *et al*., 1992) cases of taeniasis, providing one possible explanation for these findings.

All taeniasis cases for which the tapeworm species was differentiated were found to be *T. solium*. A taeniasis survey undertaken in a different region of Madagascar by Rahantamalala *et al*. (2022) also identified only cases of *T. solium* infection. To date, there has been no confirmed case of *T. saginata* taeniasis described from Madagascar.

**Figure 4.**
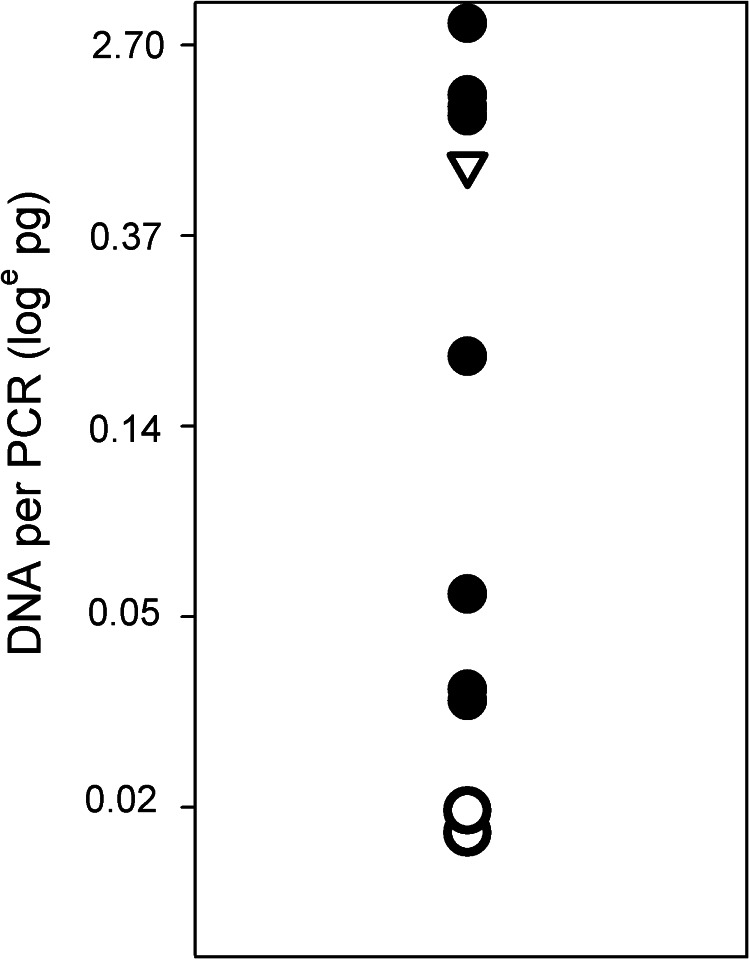
Relationship between the quantity of DNA isolated from 12 individual, egg-positive fecal samples and the outcome of *rrn*S PCR for taeniasis. Closed symbols: PCR-positive samples; open symbols: PCR-negative samples; open circles: samples positive by coproantigen test; open triangle: samples negative by PCR and coproantigen tests.

Analysis of the quantity of DNA obtained from the feces of 12 egg-positive samples revealed that the 4 samples with the smallest quantity of DNA were all negative in *rrn*S PCR utilizing 2 μL fecal DNA. Increasing the quantity of DNA to 20 μL led to 2 of these 4 samples being detected as PCR-positive. The relationship between the quantity of DNA from egg-positive samples used in *rrn*S PCR and the outcome of the test is shown in Fig. 4. One sample which was negative by both PCR and coproantigen tests was not among those with very low quantities of DNA.

We were unable to replicate the sensitivity of the *Tso31*-nested PCR described by Mayta *et al*. (2008) who recorded the test to have a sensitivity with purified *T. solium* genomic DNA as low as 200 fg, whereas in our case the test was positive at 500 fg but negative at 200 fg (Fig. 1). Variations in DNA quantitation methods are one possible reason to explain this difference. Direct comparison of the *rrn*S and *Tso31*-nested PCRs with dilutions of fecal DNA from proven cases of *T. solium* taeniasis (proven by egg detection followed by *rrn*S PCR and DNA sequencing) found that the *rrn*S PCR was superior in sensitivity to *Tso31*-nested PCR in identifying cases of taeniasis (Fig. 2). As the quantity of fecal DNA decreased, the quantity of *rrn*S PCR-amplified DNA product declined but remained detectable. However, with the *Tso31*-nested PCR, DNA products were either abundant or completely absent, even when the *rrn*S PCR remained positive. While the *Tso31* PCR involved a pair of nested PCRs, the target (*Tso31*) is a single-copy gene in the genome (Mayta *et al*., 2007). The *rrn*S PCR targets a segment of the mitochondrial genome. It is unclear just how many copies of the mitochondrial genome are there per cell in *T. solium.* Depending on cell type, human cells contain between 100 and 10 000 copies of the mitochondrial genome (Wai *et al*., 2010). Ultrastructural investigations clearly identify multiple mitochondria in *T. solium* cells (Willms *et al*., 2003) and those of other taeniids (Willms *et al*., 2003; Jabbar *et al*., 2010). The higher copy number of mitochondrial DNA targets for the *rrn*S PCR may explain the greater sensitivity of this technique with fecal DNA extracts from *T. solium* tapeworm carriers compared with the *Tso31*-nested PCR, despite the latter being a nested PCR which could have been expected to have greater analytical sensitivity. With a single sample of fecal DNA from a case of *T. asiatica* taeniasis, the *Tso31* PCR amplified a product of the same size as that obtained with *T. solium*, the sequence of which was similar to the sequence of the *Tso31* gene in *T. solium* (Fig. 1), suggesting that the *Tso31*-nested PCR may not be species-specific where *T. solium* and *T. asiatica* are sympatric.

Many PCR-based methods have been described for the diagnosis of *T. solium* taeniasis using fecal DNA; however, few have been validated using parasitologically proven cases of infection (Lightowlers *et al*., 2016). Of those that were well validated (e.g. Yamasaki *et al*., 2004; Mayta *et al*., 2008; Praet *et al*., 2013; Rodriguez-Hidalgo *et al*., 2015), they include procedures such as restriction fragment length polymorphism on the PCR amplicons (e.g. Rodriguez-Hidalgo *et al*., 2015) or quantitative PCR (qPCR) (e.g. Praet *et al*., 2013; Gordon *et al*., 2015; Ng-Nguyen *et al*., 2017). A comparison has not been made previously between the performance of different PCR-based methodologies using the same fecal DNA samples from confirmed cases of *T. solium* taeniasis. Rahantamalala *et al*. (2022) used an approach to species-specific diagnosis of taeniasis involving PCR with non-specific *cox1* primers followed by sequencing the DNA product in positive reactions, similar to the one we used. An advantage of the methodology used here, which targeted mitochondrial *rrn*S, is the small size of the target DNA sequence (267 bp) and that the 3 taeniid species infecting humans can be differentiated simply and unequivocally due to there being numerous sequence differences between the species across a region of only 30 bp. These differences can be determined from single-strand sequencing and without necessarily using sequence comparison software.

Full transmission of *T. solium* only occurs where pigs have access to materials contaminated with human feces. For this reason, transmission is limited to poor communities in developing countries. It is in these regions and countries where accurate diagnostic methods are needed for *T. solum* taeniasis. Techniques such as qPCR are poorly suited to these places. Coproantigen testing could offer a simple, potentially inexpensive diagnostic method; however, the currently used methods are neither species-specific nor are the reagents available commercially. A publication has described a species-specific coproantigen test (Parkhouse *et al*., 2020), which was evaluated with feces from 2 cases of *T. solium* taeniasis and 5 cases of *T. saginata* taeniasis, among other samples; however, there have been no further data published about this test in the ensuing 3 years since its description.

The data presented here suggest that a suitable method for species-specific diagnosis of *T. solium* taeniasis in fecal samples, which may be relatively simple and suitable for adoption in endemic countries, is an evaluation of fecal samples using a non-specific method such as egg detection or, where available, coproantigen testing, followed by a method where the positive samples are confirmed and speciated by *rrn*S PCR and DNA sequencing.

## Data Availability

Data supporting results are provided within the article.

## References

[ref1] Allan JC, Avila G, Garcia Noval J, Flisser A and Craig PS (1990) Immunodiagnosis of taeniasis by coproantigen detection. Parasitology 101, 473–477. 2092303 10.1017/s0031182000060686

[ref2] Allan JC, Craig PS, Garcia Noval J, Mencos F, Liu D, Wang Y, Wen H, Zhou P, Stringer R, Rogan M and Zeyhle E (1992) Coproantigen detection for immunodiagnosis of echinococcosis and taeniasis in dogs and humans. Parasitology 104, 347–356. 1594298 10.1017/s0031182000061801

[ref3] Ash A, Okello A, Khamlome B, Inthavong P, Allen J and Thompson RC (2017) Controlling *Taenia solium* and soil transmitted helminths in a northern Lao PDR village: impact of a triple dose albendazole regime. Acta Tropica 174, 171–178. 26001973 10.1016/j.actatropica.2015.05.018

[ref4] Deplazes P, Gottstein B, Stingelin Y and Eckert J (1990) Detection of *Taenia hydatigena* copro-antigens by ELISA in dogs. Veterinary Parasitology 36, 91–103. 2382393 10.1016/0304-4017(90)90097-u

[ref5] Garcia HH, Gonzalez AE and Gilman RH (2020) *Taenia solium* cysticercosis and its impact in neurological disease. Clinical Microbiology Reviews 33, 1–23. doi: 10.1128/CMR.00085-19 PMC7254859 32461308

[ref6] Gauci CG, Ayebazibwe C, Nsadha Z, Rutebarika C, Poudel I, Sah K, Singh DK, Stent A, Colston A, Donadeu M and Lightowlers MW (2019) Accurate diagnosis of lesions suspected of being caused by *Taenia solium* in body organs of pigs with naturally acquired porcine cysticercosis. PLoS Neglected Tropical Diseases 13, e0007408. 31237878 10.1371/journal.pntd.0007408 PMC6592510

[ref7] Gordon CA, McManus DP, Acosta LP, Olveda RM, Williams GM, Ross AG, Gray DJ and Gobert GN (2015) Multiplex real-time PCR monitoring of intestinal helminths in humans reveals widespread polyparasitism in Northern Samar, the Philippines. International Journal for Parasitology 45, 477–483. 25858090 10.1016/j.ijpara.2015.02.011

[ref8] Jabbar A, Crawford S, Mlocicki D, Swiderski ZP, Conn DB, Jones MK, Beveridge I and Lightowlers MW (2010) Ultrastructural reconstruction of *Taenia ovis* oncospheres from serial sections. International Journal for Parasitology 40, 1419–1431. 20478311 10.1016/j.ijpara.2010.04.011

[ref9] Katz N, Chaves A and Pellegrino J (1972) A simple device for quantitative stool thick-smear technique in *Schistosomiasis mansoni*. Revista do Instituto de Medicina Tropical de Sao Paulo 14, 397–400. 4675644

[ref10] Levine MZ, Lewis MM, Rodriquez S, Jimenez JA, Khan A, Lin S, Garcia HH, Gonzales AE, Gilman RH and Tsang VC (2007) Development of an enzyme-linked immunoelectrotransfer blot (EITB) assay using two baculovirus expressed recombinant antigens for diagnosis of *Taenia solium* taeniasis. Journal of Parasitology 93, 409–417. 17539427 10.1645/GE-938R.1

[ref11] Lightowlers MW (2013) Control of *Taenia solium* taeniasis/cysticercosis: past practices and new possibilities. Parasitology 140, 1566–1577. 23947762 10.1017/S0031182013001005

[ref12] Lightowlers MW, Garcia HH, Gauci CG, Donadeu M and Abela-Ridder B (2016) Monitoring the outcomes of interventions against *Taenia solium*: options and suggestions. Parasite Immunology 38, 158–169. 26538513 10.1111/pim.12291 PMC4819694

[ref13] Mayta H, Hancock K, Levine MZ, Gilman RH, Farfan MJ, Verastegui M, Lane WS, Garcia HH, Gonzalez AE, Tsang VC and Cysticercosis Working Group in Peru (2007) Characterization of a novel *Taenia solium* oncosphere antigen. Molecular and Biochemical Parasitology 156, 154–161. 17850901 10.1016/j.molbiopara.2007.07.017 PMC2082053

[ref14] Mayta H, Gilman RH, Prendergast E, Castillo JP, Tinoco YO, Garcia HH, Gonzalez AE, Sterling CR and Cysticercosis Working Group in Peru (2008) Nested PCR for specific diagnosis of *Taenia solium* taeniasis. Journal of Clinical Microbiology 46, 286–289. 17989190 10.1128/JCM.01172-07 PMC2224258

[ref15] Mwape KE, Phiri IK, Praet N, Muma JB, Zulu G, Van den Bossche P, de Deken R, Speybroeck N, Dorny P and Gabriel S (2012) *Taenia solium* infections in a rural area of Eastern Zambia – a community based study. PLoS Neglected Tropical Diseases 6, e1594. 22479664 10.1371/journal.pntd.0001594 PMC3313923

[ref16] Ng-Nguyen D, Stevenson MA, Dorny P, Gabriel S, Vo TV, Nguyen VT, Phan TV, Hii SF and Traub RJ (2017) Comparison of a new multiplex real-time PCR with the Kato Katz thick smear and copro-antigen ELISA for the detection and differentiation of *Taenia* spp. in human stools. PLoS Neglected Tropical Diseases 11, e0005743. 28686662 10.1371/journal.pntd.0005743 PMC5517074

[ref17] Parkhouse RME, Rojas RG, Aguilar CM, Medina C, Ferrer E and Cortez Alcovedes MM (2020) Diagnosis of taeniosis in rural Venezuelan communities: preliminary characterization of a *Taenia solium* specific monoclonal (VP-1) coproantigen ELISA. Acta Tropica 207, 105445. 32224076 10.1016/j.actatropica.2020.105445

[ref18] Praet N, Verweij JJ, Mwape KE, Phiri IK, Muma JB, Zulu G, van Lieshout L, Rodriguez-Hidalgo R, Benitez-Ortiz W, Dorny P and Gabriel S (2013) Bayesian modelling to estimate the test characteristics of coprology, coproantigen ELISA and a novel real-time PCR for the diagnosis of taeniasis. Tropical Medicine and International Health 18, 608–614. 10.1111/tmi.12089 23464616

[ref19] Rahantamalala A, Rakotoarison RL, Rakotomalala E, Rakotondrazaka M, Kiernan J, Castle PM, Hakami L, Choi KS, Rafalimanantsoa AS, Harimanana A, Wright PD, Lapierre SG, Schoenhals M, Small PM, Marcos LA and Vigan-Womas I (2022) Prevalence and factors associated with human *Taenia solium* taeniosis and cysticercosis in twelve remote villages of Ranomafana rainforest, Madagascar. PLoS Neglected Tropical Diseases 16, e0010265. 35404983 10.1371/journal.pntd.0010265 PMC9064101

[ref20] Ramiandrasoa NS, Ravoniarimbinina P, Solofoniaina AR, Andrianjafy Rakotomanga IP, Andrianarisoa SH, Molia S, Labouche AM, Fahrion AS, Donadeu M, Abela-Ridder B and Rajaonatahina D (2020) Impact of a 3-year mass drug administration pilot project for taeniasis control in Madagascar. PLoS Neglected Tropical Diseases 14, e0008653. 32946447 10.1371/journal.pntd.0008653 PMC7500903

[ref21] Rodriguez-Hidalgo R, Geysen D, Benitez-Ortiz W, Dorny P, Saa L and Brandt J (2015) *Improved PCR-RFLP assay for the detection and differentiation between Taenia solium and Taenia saginata in faecal samples*. Retrieved from Research Gate website: https://www.researchgate.net/profile/Richar-Rodriguez-Hidalgo/publication/278966245_Improved_PCR-RFLP_assay_for_the_detection_and_differentiation_between_Taenia_solium_and_Taenia_saginata_in_faecal_samples/links/5588609f08ae347f9bda9b6f/Improved-PCR-RFLP-assay-for-the-detection-and-differentiation-between-Taenia-solium-and-Taenia-saginata-in-faecal-samples.pdf (accessed 8 June 2023).

[ref22] Tembo A and Craig PS (2015) *Taenia saginata* taeniosis: copro-antigen time-course in a voluntary self-infection. Journal of Helminthology 89, 612–619. 24945107 10.1017/S0022149X14000455

[ref23] Trachsel D, Deplazes P and Mathis A (2007) Identification of taeniid eggs in the faeces from carnivores based on multiplex PCR using targets in mitochondrial DNA. Parasitology 134, 911–920. 17288631 10.1017/S0031182007002235

[ref24] Wai T, Ao A, Zhang X, Cyr D, Dufort D and Shoubridge EA (2010) The role of mitochondrial DNA copy number in mammalian fertility. Biology of Reproduction 83, 52–62. 20130269 10.1095/biolreprod.109.080887 PMC2888963

[ref25] Willms K, Robert L and Caro JA (2003) Ultrastructure of smooth muscle, gap junctions and glycogen distribution in *Taenia solium* tapeworms from experimentally infected hamsters. Parasitology Research 89, 308–316. 12632169 10.1007/s00436-002-0733-1

[ref26] World Helath Organization (2015) *Investing to overcome the global impact of neglected tropical diseases*. Third WHO report on neglected tropical diseases. WHO/HTM/NTD/2015.1. Geneva, Switzerland: World Health Organization.

[ref27] Yamasaki H, Allan JC, Sato MO, Nakao M, Sako Y, Nakaya K, Qiu D, Mamuti W, Craig PS and Ito A (2004) DNA differential diagnosis of taeniasis and cysticercosis by multiplex PCR. Journal of Clinical Microbiology 42, 548–553. 14766815 10.1128/JCM.42.2.548-553.2004 PMC344500

